# Supplementary material for: Associability-modulated loss learning is increased in posttraumatic stress disorder
Source: eLife. 2018 Jan 9;7:e30150. doi: 10.7554/eLife.30150 (PMC5760201; doi:10.7554/eLife.30150)
Supplement: Supplementary file 1. — (A) Table 1A. Demographic and clinical characteristics of veteran sample (N = 68). Table 1A legend: amean ± SE; t-test; bnumber (%); χ2 test. CAPS: Clinician Administered PTSD Scale; BDI: Beck Depression Inventory; CES: Combat Exposure Scale; WTAR: Wechsler Test of Adult Reading. (B) Table 1B. fMRI activation clusters for loss associability independent of covariates of PTSD, BDI, CES, age, and gender (intercept term; associated with Figure 3d). (C) Table 1C. fMRI activation clusters for loss associability correlation with PTSD diagnosis; covariates of BDI, CES, age, and gender (associated with Figure 4b). Table 1C legend: Italicized peaks are local maxima in separate anatomical regions > 4 mm apart. (D) Table 1D. fMRI activation clusters for loss associability correlation with PTSD symptom severity (total CAPS score); covariates of BDI, CES, age, and gender (associated with Figure 5a). Table 1D legend: Italicized peaks are local maxima in separate anatomical regions > 4 mm apart. (E) Table 1E. fMRI activation clusters for loss associability correlation with hyperarousal; covariates of BDI, CES, age, and gender (associated with Figure 5a). (F) Table 1F. fMRI activation clusters for loss associability correlation with avoidance/numbing; covariates of BDI, CES, age, and gender (associated with Figure 5a). Table 1F legend: Italicized peaks are local maxima in separate anatomical regions > 4 mm apart. (G) Table 1G. fMRI activation clusters for loss associability correlation with re-experiencing; covariates of BDI, CES, age, and gender (associated with Figure 5a). (H) Table 1H. fMRI activation clusters for loss associability correlation with PTSD diagnosis with associability value entered as a second parametric modulator after prediction error; covariates of BDI, CES, age, and gender (associated with Figure 4b). (I) Table 1I. fMRI activation clusters for loss associability correlation with PTSD diagnosis; covariates of BDI, CES, age, and gender, thresholded FWE p<0.05 usi [file elife-30150-supp1.docx]

**Supplementary File 1. Supplementary Tables 1A through 1I**

**Supplementary File 1A. Table 1A.**

| **Measure** | **Veteran Controls** | **Veterans with PTSD** | **Group Comparison** |
| --- | --- | --- | --- |
| **PTSD Severity (CAPS)^a^** | 12.6 ± 2.53 | 66.7 ± 4.27 | t_67_ = -13.01, p < .001 |
| **Depressive Symptoms (BDI)^a^** | 9.38 ± 1.83 | 24.6 ± 2.18 | t_67_ = -5.19, p < .001 |
| **Combat Exposure (CES)^a^** | 16.0 ± 1.82 | 21.9 ± 1.35 | t_65_ = -3.69, p < .01 |
| **Estimated IQ (WTAR)^a^** | 113.5 ± 1.61 | 105.2 ± 1.53 | t_67_ = 3.73, p < .001 |
| **Psychotropic medication^b^** | 0 (0%) | 20 (62%) | χ^2^_1_ = 20.1, p < .001 |
| **Age^a^** | 33.3 ± 1.77 | 32.3 ± 1.21 | t_67_ = 0.51, p > .1 |
| **Gender (female)^b^** | 3 (10.3%) | 5 (12.8%) | χ^2^_1_ = .0001, p > .1 |

**Supplementary File 1B. Table 1B.**

| **Cluster Number** | **Region** | **Peak MNI Coordinate** | | | **Peak T Value** | **Cluster Size** |
| --- | --- | --- | --- | --- | --- | --- |
| 1 | Right parietal lobe | 46 | -56 | 44 | 9.29 | 4706 |
| 2 | Left cerebellum | -34 | -68 | -50 | 8.57 | 13990 |
| 3 | Right middle frontal gyrus | 38 | 0 | 54 | 8.24 | 9853 |
| 4 | Left parietal lobe | -38 | -52 | 40 | 7.86 | 3113 |
| 5 | Right postcentral gyrus | 20 | -44 | 72 | -6.54 | 1971 |
| 6 | Right calcarine sulcus | 18 | -46 | 4 | -6.45 | 562 |
| 7 | Left calcarine sulcus | -26 | -46 | 0 | -6.45 | 1100 |
| 8 | Right superior temporal gyrus | 54 | -28 | 14 | -6.45 | 2919 |
| 9 | Right thalamus | 12 | -6 | 10 | 5.9 | 2039 |
| 10 | Right superior occipital lobe | -26 | -96 | 34 | -5.7 | 1277 |
| 11 | Posterior cingulate | 0 | -36 | 26 | 5.69 | 407 |
| 12 | Left parietal lobe | -18 | -56 | 64 | -5.65 | 870 |
| 13 | Left orbitofrontal cortex | -8 | 25 | -12 | -5.49 | 874 |
| 14 | Left posterior cingulate | -16 | -36 | 40 | -5.41 | 203 |
| 15 | Left insula | -42 | -16 | 2 | -5.34 | 1445 |
| 16 | Left middle frontal gyrus | -40 | 52 | -2 | 5.3 | 1507 |
| 17 | Left inferior frontal gyrus | -52 | 6 | 40 | 5.25 | 446 |
| 18 | Left precentral gyrus | -32 | -4 | 58 | 4.91 | 411 |
| 19 | Right middle occipital gyrus | 52 | -90 | 14 | -4.69 | 271 |

**Supplementary File 1C. Table 1C.**

| **Cluster Number** | **Region** | **Peak MNI Coordinate** | | | **Peak T Value** | **Cluster Size** |
| --- | --- | --- | --- | --- | --- | --- |
| 1 | Left precentral gyrus | -54 | -4 | 50 | 6.5 | 418 |
| 2 | Right rolandic operculum | 54 | 8 | 50 | 6.44 | 5346 |
|  | *Right insula* | *30* | *18* | *-14* | *6.04* |  |
| 3 | Left insula | -28 | 14 | 12 | 5.47 | 2643 |
| 4 | Right precentral gyrus | 48 | -8 | 54 | 4.99 | 308 |
| 5 | Right middle frontal gyrus | 32 | 38 | 36 | 4.64 | 409 |
|  | *Right superior frontal gyrus* | *24* | *46* | *40* | *4.18* |  |
| 6 | Left superior frontal gyrus | 2 | 42 | 42 | 4.38 | 244 |
| 7 | Left middle frontal gyrus | -26 | 54 | 30 | 4.38 | 241 |
|  | *Left inferior frontal gyrus* | *-32* | *38* | *14* | *4.15* |  |

**Supplementary File 1D. Table 1D.**

| **Cluster Number** | **Region** | **Peak MNI Coordinate** | | | **Peak T Value** | **Cluster Size** |
| --- | --- | --- | --- | --- | --- | --- |
| 1 | Right inferior frontal gyrus | 20 | 8 | -16 | 5.21 | 545 |
| 2 | Right middle temporal gyrus | 54 | -20 | -8 | 5.14 | 666 |
|  | *Right superior temporal gyrus* | *52* | *-28* | *6* | *3.99* |  |
| 3 | Left precentral gyrus | -54 | 2 | 48 | 4.73 | 254 |
| 4 | Right rolandic operculum | 54 | 8 | 2 | 4.72 | 263 |
|  | *Right inferior frontal gyrus* | *52* | *6* | *18* | *4.08* |  |
| 5 | Right thalamus | 14 | -6 | -8 | 4.65 | 220 |
| 6 | Left putamen | -14 | 6 | -14 | 4.35 | 278 |
| 7 | Right medial frontal gyrus | 14 | -18 | 56 | 4.28 | 249 |
|  | *Right superior frontal gyrus* | *24* | *-10* | *76* | *4.22* |  |

**Supplementary File 1E. Table 1E.**

| **Cluster Number** | **Region** | **Peak MNI Coordinate** | | | **Peak T Value** | **Cluster Size** |
| --- | --- | --- | --- | --- | --- | --- |
| 1 | Right inferior frontal gyurs | 36 | 20 | -18 | 5.01 | 268 |

**Supplementary File 1F. Table 1F.**

| **Cluster Number** | **Region** | **Peak MNI Coordinate** | | | **Peak T Value** | **Cluster Size** |
| --- | --- | --- | --- | --- | --- | --- |
| 1 | Right fusiform gyrus | 52 | -32 | -26 | 5.86 | 2716 |
|  | *Right inferior frontal gyrus* | *20* | *8* | *-16* | *5.18* |  |
| 2 | Left superior temporal gyrus | -40 | -30 | 8 | 5.8 | 1759 |
|  | *Left insula* | *-36* | *-18* | *18* | *4.96* |  |
| 3 | Left precentral gyrus | -54 | 4 | 50 | 4.91 | 299 |
| 4 | Superior frontal gyrus | 12 | -4 | 78 | 4.8 | 262 |
| 5 | Left cerebellum | -40 | -46 | -48 | 4.68 | 270 |

**Supplementary File 1G. Table 1G.**

| **Cluster Number** | **Region** | **Peak MNI Coordinate** | | | **Peak T Value** | **Cluster Size** |
| --- | --- | --- | --- | --- | --- | --- |
| 1 | Right superior temporal gyrus | 58 | -22 | -4 | 4.93 | 296 |

**Supplementary File 1H. Table 1H.**

| **Cluster Number** | **Region** | **Peak MNI Coordinate** | | | **Peak T Value** | **Cluster Size** |
| --- | --- | --- | --- | --- | --- | --- |
| 1 | Right inferior frontal gyrus | 22 | 26 | -18 | 5.47 | 2413 |
| 2 | Left putamen | -32 | -2 | -8 | 4.66 | 655 |
| 3 | Right insula | 52 | 8 | 2 | 4.83 | 320 |
| 4 | Left precentral gyrus | -58 | -2 | 48 | 5.36 | 296 |
| 5 | Left superior temporal gyrus | -44 | -28 | 4 | 4.79 | 250 |
| 6 | Right middle frontal gyrus | 34 | 38 | 30 | 4.15 | 229 |

**Supplementary File 1I. Table 1I.**

| **Cluster Number** | **Region** | **Peak MNI Coordinate** | | | **Peak T Value** | **Cluster Size** |
| --- | --- | --- | --- | --- | --- | --- |
| 1 | Left precentral gyrus | -54 | -4 | 50 | 6.5 | 50 |
| 2 | Right rolandic operculum | 54 | 8 | 50 | 6.44 | 40 |
| 3 | Right insula | 30 | 18 | -14 | 6.04 | 7 |
| 4 | Right inferior frontal gyrus | 20 | 10 | -16 | 5.64 | 3 |
| 5 | Right inferior frontal gyrus | 22 | 26 | -18 | 5.56 | 1 |
| 6 | Left insula | -28 | 14 | 12 | 5.47 | 2 |
| 7 | Right inferior frontal gyrus | 50 | 4 | 22 | 5.40 | 1 |
